# Perspectives on Multiscale Colloid-Based Materials for Biomedical Applications

**DOI:** 10.1021/acs.langmuir.3c01274

**Published:** 2023-09-21

**Authors:** Wen Li, Judah Huberman-Shlaes, Bozhi Tian

**Affiliations:** † Department of Chemistry, The University of Chicago, Chicago, Illinois 60637, United States; ‡ Department of Biology, The University of Chicago, Chicago, Illinois 60637, United States; § The James Franck Institute, The University of Chicago, Chicago, Illinois 60637, United States; ∥ The Institute for Biophysical Dynamics, The University of Chicago, Chicago, Illinois 60637, United States

## Abstract

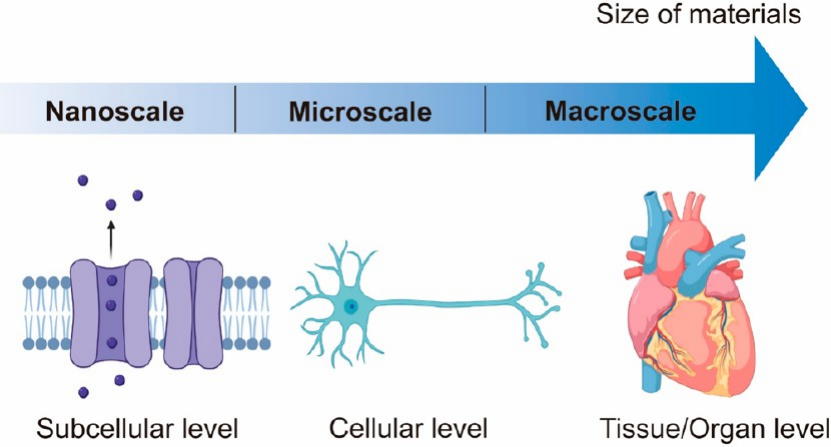

Colloid-based materials with tunable biophysical and chemical properties have demonstrated significant potential in a wide range of biomedical applications. The ability to manipulate these properties across various size scales, encompassing nano-, micro-, and macrodomains, is essential to enhancing current biomedical technologies and facilitating the development of novel applications. Focusing on material design, we explore various synthetic colloid-based materials at the nano- and microscales and investigate their correlation with biological systems. Furthermore, we examine the utilization of the self-assembly of colloids to construct monolithic and macroscopic materials suitable for biointerfaces. By probing the potential of spatial imaging and localized drug delivery, enhanced functionality, and colloidal manipulation, we highlight emerging opportunities that could significantly advance the field of colloid-based materials in biomedical applications.

## Introduction

Colloidal systems, also known as colloids, are a state of subdivision such that the molecules or polymolecular particles dispersed in a medium at least one dimension between approximately 1 nm and 1 μm or that system discontinuities are found at distances on that order.^1^ Colloids can be classified based on the nature of the dispersed phase and the continuous phase.^2^ Some common types of colloidal systems include a sol (solid is dispersed in liquid), gel (liquid is dispersed in solid), aerosol (solid is dispersed in gas), foam (gas is dispersed in solid or liquid), and emulsion (liquid is dispersed in another immiscible liquid). Colloidal systems are important for targeted biological interactions because they can fit the natural biological liquid environment while allowing for precise control of certain properties.

Naturally occurring colloidal systems play a crucial role in many biological activities. Examples of colloidal systems in biology include proteins, lipids, nucleic acids, and polysaccharides, which can form complex structures, such as micelles, liposomes, and hydrogels. These colloidal systems provide a suitable environment for many biological reactions to occur as well as transport and storage of important molecules within cells. One important example of colloidal systems in biology is the cytoplasm of cells containing various proteins, lipids, and nucleic acids that provide a suitable environment for many biological reactions. Proteins in the cytoplasm are able to form colloidal structures that enable them to carry out enzymatic reactions and molecular recognition processes.^3^ For instance, the ribosome is a complex colloidal assembly that plays a crucial role in protein synthesis. It consists of RNA and protein components that form a highly organized structure, allowing it to catalyze the formation of peptide bonds between amino acids.^4^ Another important example is blood, which is a complex mixture of cells, proteins, lipids, and electrolytes, many of which exist in colloidal form. The wide variety of colloidal proteins in blood helps maintain proper fluid balance^5^ and plays important roles in immune function^6^ and blood clotting.^7^ Red blood cells, which exist in colloidal form, can carry oxygen to tissues. Other components of blood that exist in colloidal forms include lipoproteins and platelets. The study of the colloidal system in blood is essential for understanding many physiological processes and has important implications for the diagnosis and treatment of many diseases.

Since colloidal systems are so significant for biological activities, scientists have synthesized many colloidal particles for investigating biological activities or treating diseases. Synthetic nanoparticle vaccines, containing nucleic acid or protein, can penetrate the mucus barrier and directly stimulate the antigen-specific T cells through antigen and antibody interaction, triggering the downstream biological response.^8,9^ Beyond the direct chemical stimulation, in order to have a better manipulation and spatial control over the synthetic colloid system, scientists use a variety of external stimulation, including optical, magnetic, electrical, and acoustic stimulations.^10^ Optical stimulation can be utilized via the photoelectrical effect of semiconductor-based biointerfaces, such as silicon nanowires, which utilize light-induced stimulation for nongenetic modulation.^11^ Similarly, quantum dots can rely on near-infrared light (NIR) for biological stimulation, allowing for deep tissue neural modulation and communication.^12^ Along with the photoelectric effect, colloidal systems can utilize the photothermal effect for biomodulation. For example, nanoparticles can embed a photothermal dye. Upon stimulation with NIR, these nanoparticles generate heat, which can be utilized for a variety of biological applications, including muscle contraction and the death of cancer cells.^13^ Additionally, by binding magnetic nanoparticles to the surfaces of cells, a magnetic field can be utilized to manipulate and control cell function, which provides a way to isolate and explore cellular mechanics and ion channel activation, and can be applied in tissue engineering and regenerative medicine.^14^ Acoustic stimulation offers an opportunity to noninvasively stimulate the deep tissue with sharp spatial resolution. Nanoscale piezoelectric materials can locally activate voltage-gated ion channels by converting acoustic waves to electric fields. The use of tetragonal barium titanate nanoparticles as wireless nanotransducers is able to elicit a significant cellular response for SH-SY5Y neuron-like cells in term of calcium and sodium fluxes.^15^

**Figure 1:**
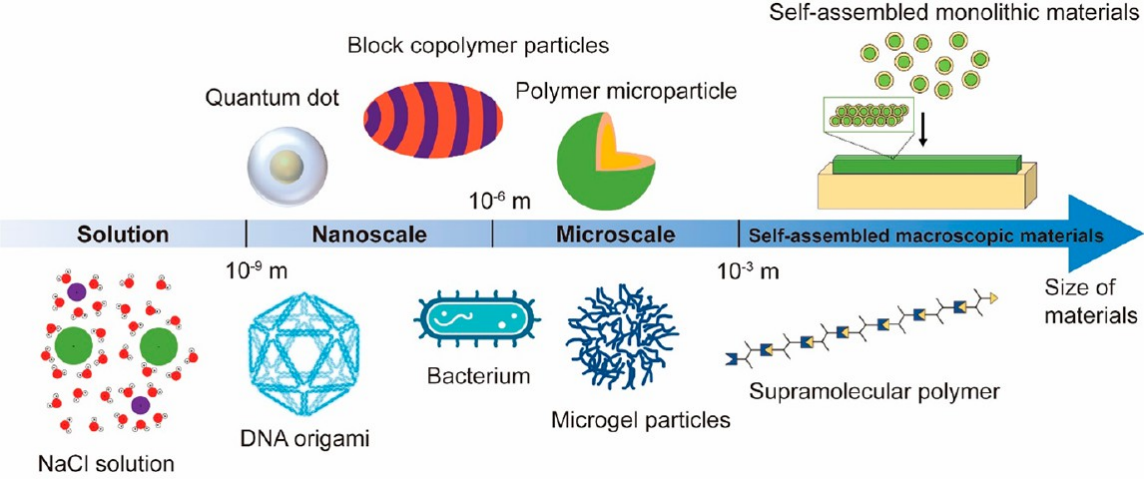
Size spectrum showing solution (<1 nm) and various colloid-based materials. Created with BioRender.com.

Herein, we discuss colloid-based synthetic materials at different scales containing colloidal nanoparticles, microparticles, and macroscopic materials formed by the self-assembly of colloidal particles (Figure 1). This size variability imparts unique properties and functionalities to colloid-based materials, enabling their application in diverse biointerfaces. At the nanoscale, colloids exhibit remarkable features that facilitate novel applications in biotechnology and medicine. Block copolymers particles, nanoscale colloidal systems, are composed of two or more distinct polymer chains covalently linked together. Their ability to self-assemble into nanostructures with various morphologies enables their application in drug delivery and release.^16^ DNA origami, a technique that involves the folding of a long, single-stranded DNA molecule into intricate nanostructures, can be employed in the design of biophysical tools, drug delivery, and biological imaging.^17^ Transitioning to the microscale, colloidal systems can be found in biological entities such as cells and bacteria as well as synthetic materials such as microgel particles and polymer microparticles. These microscale colloidal systems are critical in numerous biotechnological applications. For instance, microgel particles can be used to encapsulate and release drugs, proteins, or other bioactive molecules in a controlled manner.^18^ Polymer microparticles can serve as carriers for bioactive agents, encapsulating cells and proteins, or particle sensors with tailored functionalities.^19^ Finally, on the macroscopic scale, colloid-based materials can be created through the self-assembly of small nanoparticle building blocks, resulting in materials with hierarchical structures and unique properties.^20^ These macroscopic colloid-based materials can be employed in the development of advanced biointerfaces, such as tissue scaffolds^21^ and brain–machine interfaces.^22^ In summary, colloid-based materials spanning various size scales, from nanoscale features to macroscopic structures, offer a wealth of possibilities for the development of advanced biointerfaces. Understanding the unique properties and functionalities of these systems is crucial to the design of innovative biomedical applications and technologies.

## Nanoscale Colloid-Based Materials for Biointerfaces

Biological structures in nature are complex and exhibit multiscale features. Nanoscale elements, such as lipid bilayers, ion channels, organelles, and synaptic junctions, form the foundation for biological activities, which are difficult to target precisely by macroscopic materials and devices.^23^ Nanoparticle-based materials and devices, as the smallest components of colloidal systems, possess unique strengths in forming seamless interfaces with biological system at both cellular and tissue levels, owing to their small size, controllability, functionality, and specificity.^24^ The chemical structures of synthetic nanoscale colloidal systems, such as quantum dots (QDs), micelles, and DNA origami, can be tailored to produce biomaterials that trigger different downstream biological effects. Some engineered nanoparticles can respond to external stimuli, allowing for nanoscale precision sensing and stimulation. In this section, we discuss recent advances in nanoparticle-enabled biointerfaces.

**Figure 2:**
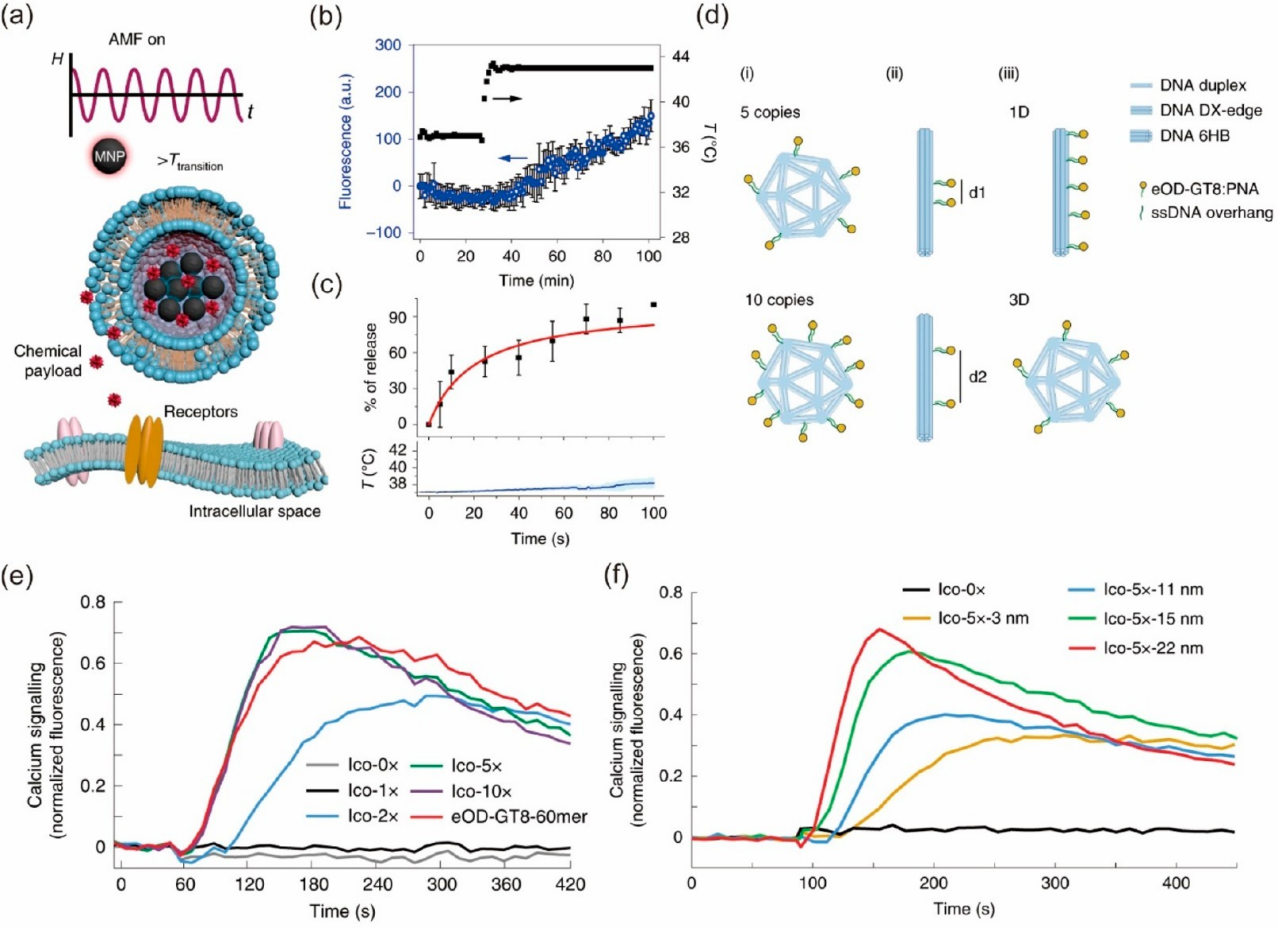
Nanoscale colloid-based materials for biointerfaces. (a) Schematic representation of the AMF-triggered chemical payload. (b) Temperature increase causes an increase in fluorescent dye release. (c) AMF-triggered dye release and the corresponding temperature values. Panels a, b and c are adapted with permission from ref (27). Published 2019 by Springer Nature. (d) Both the icosahedral and 6HB structures were used to explore (i) the stoichiometry; (ii) the interantigen distances, d1 and d2; and (iii) the 1D versus 3D dimensionality of eOD-GT8 antigen presentation (DX, double-crossover). (e) glVRC01 B cells’ calcium signaling response to DNA-NPs modified with eOD-GT8 activates IgM-BCR at 5 nM eOD-GT8. (f) Plot of Fluo-4 calcium probe fluorescence versus time following the addition of 5 nM eOD-GT8 antigen to glVRC01 B cells. Panels d, e, and f are adapted with permission from ref (29). Published 2020 by Springer Nature.

Precise delivery of neuromodulators to the brain allows for the investigation of the relationship between chemical manipulation and animal behavior.^25^ Traditional systemic injection methods, such as intravenous and intraperitoneal injections, suffer from low temporal resolution and are further impeded by the blood–brain barrier, while implanted cannulae or infusion pumps are often too invasive.^26^ A chemomagnetic gate has been developed to achieve brain neuron control with spatial and temporal precision (Figure 2a).^27^ This gate consists of thermally responsive liposomes, magnetic nanoparticles (MNPs), and neuromodulators. In the presence of alternating magnetic fields (AMFs), heat is generated, causing the release of the neuromodulators. The liposomes have a phase-transition temperature of 43 °C, ensuring that the chemical payload begins releasing at a temperature 6 °C higher than body temperature. Fluorescent dye is used as a payload to confirm the minimal background leakage at 37 °C (Figure 2b), while at 43 °C, the fluorescence intensity starts to increase, indicating the release of the chemical payload. Figure 2c shows a little temperature increase, while all of the chemical payload is released under the AMF stimulus, avoiding thermal damage to cells. This micelle-based material has been further used in animal behavior studies to investigate the influence of DRD1 agonists and antagonists on social behavior. Only the mice injected with DRD1 agonist-loaded magnetoliposomes displayed an increased social preference after the AMF stimulation.

DNA origami has emerged as a powerful method for generating DNA nanostructures with dynamic properties and nanoscale control, enabling complex biophysical studies and the fabrication of next-generation therapeutic devices.^28^ This technique offers a programmable platform for investigating the effects of antigen copy number, spacing, affinity, dimensionality, and scaffold rigidity on B-cell activation.^29^ Two structured DNA-nanoparticle variants have been used for this investigation: a three-dimensional (3D) icosahedral DNA nanoparticle with a 40-nm-diameter size and a 1D rigid-rod six-helix bundle with maximal dimensions of 80 nm. The location and number of antigens can be specified and spatially programmed to create DNA origami nanoparticles with controlled antigen copy numbers, interantigen distance, and the spatial dimensionality of antigen presentation (Figure 2d). Studies show that DNA origami nanoparticles bearing two or more copies of antigens trigger increasing cellular responses with increasing antigen valency (Figure 2e). However, the cellular response signal plateaus at a valency of five or higher. Further investigation of the influence of antigen distance on B-cell activation was conducted by arranging the distance between two antigens on a 1D rigid-rod DNA origami. As the distance between the two antigens increases, the total calcium signal increases significantly until the distance between the two antigens reaches 28 nm. Similarly, increasing interantigen distance on the DNA icosahedron also leads to an increase in cellular response (Figure 2f).

Light-driven neuron stimulations have been investigated by using gold nanoparticles,^30^ carbon nanotubes,^31^ and silicon nanostructures.^32^ However, the light used in most cases is in the range of 520–808 nm and has limited penetration through skulls and brain tissue. To achieve deeper penetration, thermal stimulation triggered by nanoparticles absorbing longer-wavelength light has been explored. Semiconducting polymer nanoparticle-based photoacoustic nanotransducers (PANs) have been designed to achieve neural stimulation with submillimeter spatial resolution and negligible heat deposition.^33^ NIR-II-absorbing semiconducting polymer bis-isoindigo-based polymer (BTII) is synthesized and is mixed with an amphiphilic polymer polystyrene-*block*-poly(acryl acid) (PS-*b*-PAA) via a nanoprecipitation method. The hydrophobic portion of PS-*b*-PAA forms π–π stacking with BTII to construct the nanoparticles, while the hydrophilic portion makes the nanoparticles soluble in water. In the photoacoustic process, the semiconducting polymer will absorb light, convert it to heat, and generate a temperature rise. Then the thermoelastic expansion takes place, resulting in the emission of acoustic waves.^34^ The negatively charged PANs have been found to bind to the neuron membrane and achieve single neuron activation, while other neurons in the field remain unchanged. The nanoparticles are further tested on a mouse. PAN solution is injected into the primary motor cortex of the mouse. Upon light illumination, the motor cortex is activated, which further invokes subsequent motor responses.

## Microscale Colloid-Based Materials for Biointerfaces

Microscale colloidal particles have garnered significant interest as versatile building blocks for the development of novel materials and devices in biointerfaces and bioelectronics due to their tunable physicochemical properties and ease of functionalization. Comprising a wide range of materials, microscale particles include nonliving components such as microgels, inorganic microparticles, polymer microparticles, and water/oil emulsions as well as living components such as bacteria, fungi, plant cells, and animal cells. Spanning sizes from several micrometers to submicrometer dimensions, these particles can be employed in biointerface applications to improve the delivery of therapeutics and modulate cell/tissue behavior. In this section, we discuss recent advances in isolated microparticle-based materials and devices for biointerface applications.

**Figure 3:**
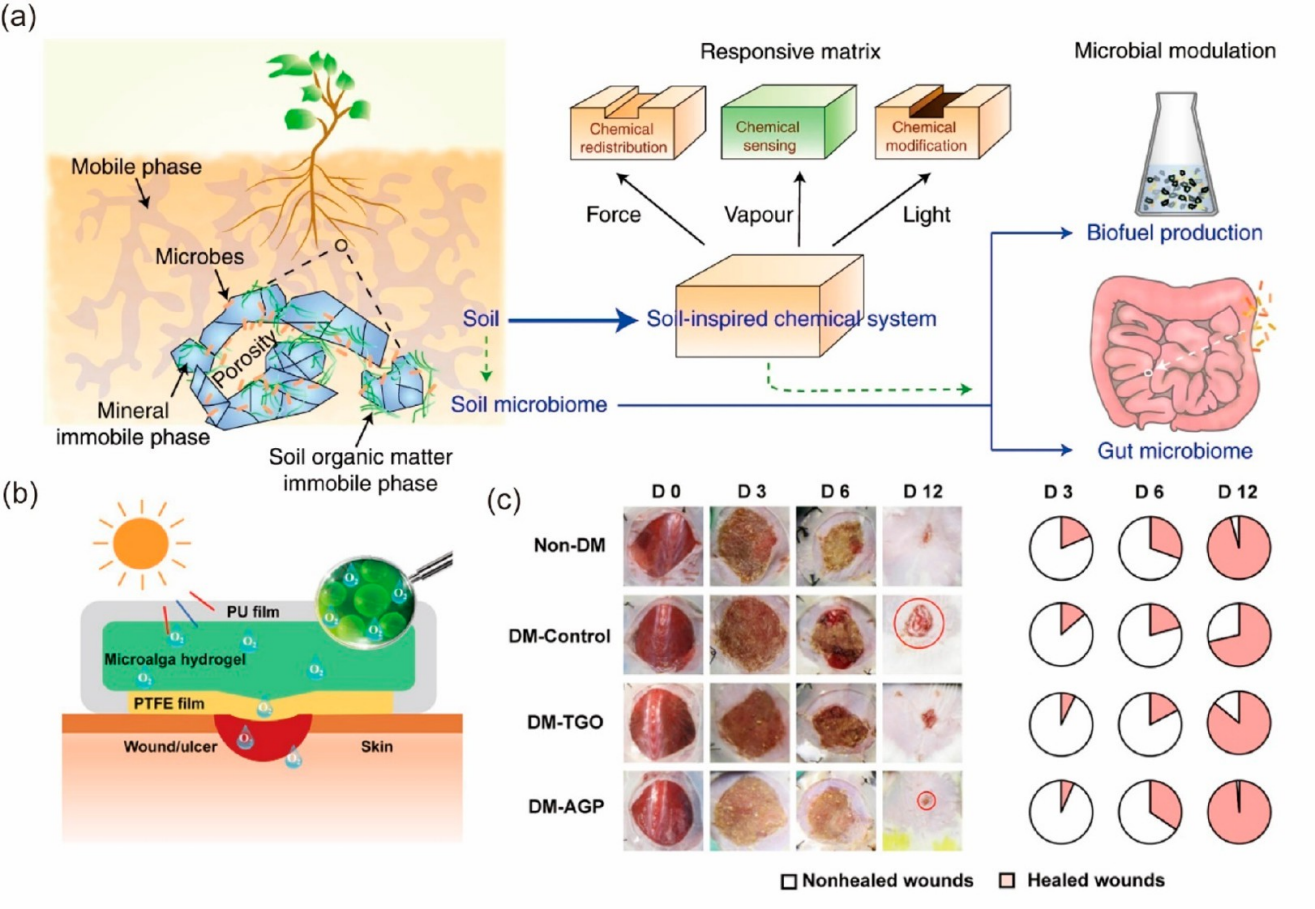
Microscale colloid-based materials for the biointerface. (a) Schematics of soil-inspired material, made up of inorganic nanoclay, organic starch, and liquid metal mobile phase layers. The soil exhibits chemical redistribution via its responsiveness to light, vapor, and force. Panel a is adapted with permission from ref (36). Published 2023 by Springer Nature. (b) Microalga-hydrogel patch (AGP) preparation (schematic illustration). (c) Wound area images on different days of treatment (days 0, 3, 6, and 12). Panels b and c are adapted from ref (40). Copyright 2020 The Authors, some rights reserved; exclusive license AAAS. Distributed under a CC BY-NC 4.0 license http://creativecommons.org/licenses/by-nc/4.0/. Reprinted with permission from AAAS.

The interaction between microbiota and their colonized environments is critical for biogeochemical cycles, ecological resilience, and human health.^35^ Inspired by the microbially colonized nature of soil, our group designed a chemical system that could serve as a responsive platform for the modulation of microbial systems (Figure 3a).^36^ The system comprises nanoclay, starch granules, and liquid-metal particles. This soil-inspired chemical system could largely enhance the growth of biofilm by 43% after lasing and promote biofuel synthesis. The biochemical impact of the soil-inspired material is also further tested on mice. Tetracycline is used to induce significant microbiome dysbiosis, and the absolute gut microbiota abundance undergoes a significant reduction. Oral administration of the soil-inspired material significantly boosted gut microbial abundance according to the LEFSe taxa analysis, which is an indicator of healthy microbiota.^37^ Dextran sulfate sodium (DSS) is further used to induce the ulcerative colitis rodent model, which is a more severe gastrointestinal condition. Soil-inspired materials and materials without one component (starch, nanoclay, liquid metal) and water (as the control group) are orally administered. Histology staining and analysis showed an improved pathological appearance of the colon. The therapeutic efficacy of the complete soil-inspired material was greater than that of material that lacked components. This work presents a direction where a nature-inspired synthetic material or chemical system could be unexpectedly useful in modulating the biointerface and improving human health.

Chronic wounds suffer from a lack of oxygen delivery, which impairs the healing process via the inhibition of important healing processes such as angiogenesis, reepithelialization, and extracellular matrix (ECM) synthesis.^38^ Sustainable and localized oxygen delivery could avoid hyperoxia and accelerate chronic wound healing. An oxygen-generating system based on oxygen-release microspheres and a reactive oxygen species-scavenging hydrogel has been developed to sustainably deliver oxygen for at least 2 weeks.^39^ The sustained release of oxygen significantly increased the wound closure rate by augmenting the survival and migration of keratinocytes and dermal fibroblasts, promoting angiogenic growth factor expression and angiogenesis in diabetic wounds and decreasing the level of proinflammatory cytokine expression. Beyond using synthetic material systems, living material has been used to supply oxygen. Natural alga can convert carbon dioxide into oxygen through photosynthesis. An alga-gel patch (AGP), made of a living microalgae hydrogel, a gas- and water-permeable PTFE film, and a gas-impermeable PU film, can consume carbonates to produce O_2_ and CO_2_ through respiration and photosynthesis (Figure 3b).^40^ Compared with topical gaseous oxygen (TGO) therapy, the AGP shows better oxygen penetration through intact mouse skin. Four groups of animals were further used to evaluate the treatment effect: diabetic mouse wounds treated with AGP (DM-AGP), diabetic mouse wounds treated with TGO therapy (DM-TGO), a diabetic mouse wound without treatment (DM-control), and a normal mouse wound (non-DM). As shown in Figure 3c, wounds treated with AGP healed significantly faster than wounds treated with TGO and diabetic control groups and are similar to normal wound healing.

Droplet microfluidic devices have been widely used for the generation, manipulation, and analysis of discrete liquid droplets within an immiscible liquid phase flowing through channels.^41^ Generally, three types of emulsions can be formed through this technology—oil-in-water (O/W), water-in-oil (W/O), and water-in-water (W/W)—each with distinct properties.^42^ O/W and W/O emulsions are the most commonly produced emulsions in microfluidic devices, and they have attracted significant attention in the field of colloidal systems due to their unique characteristics. O/W emulsions can serve as compartments for encapsulating hydrophobic molecules while retaining the ability to form a stable dispersion in water, which is important to drug delivery.^43^ In drug formulation, control of the spatial and temporal kinetics of drug release at the site of action is key to achieving an optimal pharmokinetic effect.^44,45^ By using a microfluidic method and polymerizing the monomers inside the oil droplets, size-tunable drug-loaded biodegradable polymer microparticles can be created, which can control the drug release kinetics.^44^ W/O emulsions can serve as microreactors for enzymatic reactions or incorporate living organisms. Cells can be encapsulated in W/O droplets, allowing for the isolation and confinement of individual cells, making them ideal for single-cell analysis.^46^ The droplets are able to be functionalized with biomolecules, allowing for the study of protein engineering,^47^ enzyme kinetics,^48^ and other biological process. W/O emulsions have the ability to encapsulate cells and bioactive materials, but the resulting microparticles need to be transferred from the oil phase to the water phase for biomedical use, which can lead to a delay in transfer and a subsequent reduction in cell viability.^43^ To address this issue, researchers have proposed W/W emulsion systems.^49^ These systems use aqueous solutions of two chemically dissimilar polymers, which can undergo phase separation at high concentrations. This phase separation aids in the transfer of microparticles into the water phase, thereby enhancing the cell viability.

## Self-Assembled Macroscopic Colloid-Based Materials for Biointerfaces

Real-world bioelectronics applications, including drug delivery systems, biosensing, and electrical modulation of tissues and organs, largely require such a biointerface at the macroscopic level.^50^ However, traditional macroscopic bioelectronic devices are usually rigid and mechanically invasive to cells and tissues, which also makes seamless biointerfaces difficult to form. Self-assembly features a promising way to improve the macroscopic device performance, by which components, either separate or linked, spontaneously form an ordered structure. Self-assembly can occur and form components with sizes ranging from the molecular to the macroscopic level.^51^ Macroscopic materials made from self-assembly usually possess unique properties that originate from their structures. Such macroscopic materials and devices are monolithic and can be used to modulate a large area of tissue/organ,^50^ sense special biomarkers to monitor human health,^52^ and record the electrophysiology signal.^22^

**Figure 4:**
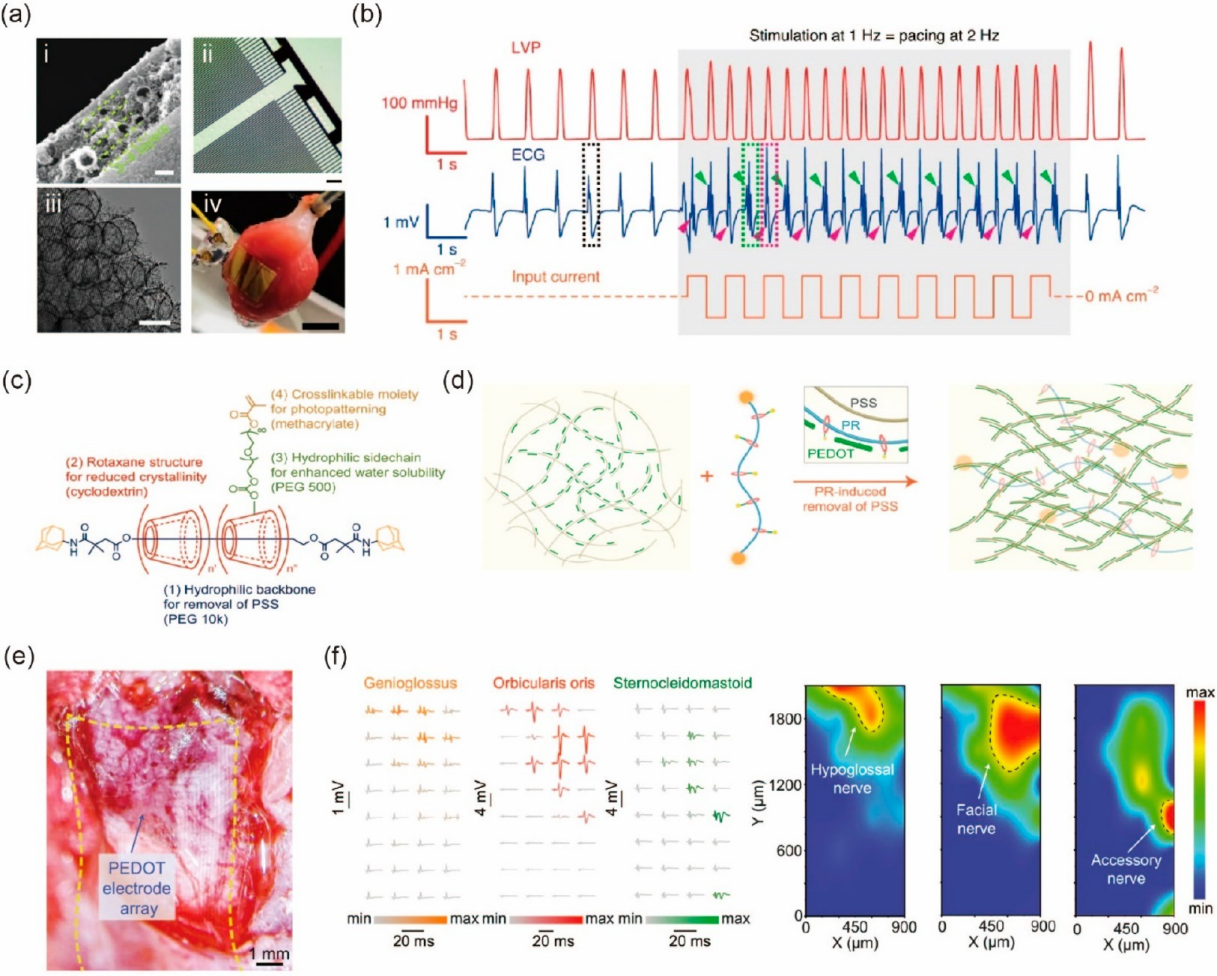
Self-assembled macroscopic colloid-based materials for biointerfaces. (a) Images of the microcapacitor material: (i) cross-sectional view and (iii) associated top view of hierarchical porous carbon; (ii) close-up view of a hierarchical porous carbon microsupercapacitor device; and (iv) view of the supercapacitor device wrapped around cardiac tissue. (b) Corresponding ECG graph and left ventricular pressure (LVP) profile for an isolated stimulated heart at 1 Hz. Panels a and b are adapted with permission from ref (50). Published 2021 by Springer Nature. (c) Chemical structures and individual roles for PR-PEGMA building blocks. (d) Schematic illustration of enhanced conductivity due to the interaction between PR and PEDOT:PSS. (e) Microscopic image of the fourth ventricle with a stretchable electrode attached. (f) Recorded muscle activities of the tongue, whisker, and neck postelectrical stimulation of the brain stem, along with corresponding activation maps based on muscle activity. Panels c, d, e and f are adapted with permission from ref (22). Published 2022 by the American Association for the Advancement of Science.

The micelle-enabled self-assembly process was used to make a binder-free, carbon-based, and flexible microsupercapacitor system.^50^ Through biphasic interaction, the triblock copolymer Pluronic F127 and resin were mixed to form nanoscale micelles. Then 200–300 nm spheres coated with dopamine were further added to the mixture. Through layer–layer spinning-coating, solvent-evaporation-induced self-assembly, carbonization, and template removal, a monolithic carbon membrane was yielded, with layered nanoporous structure and macroporous structures as shown in Figure 4a i and iii. The nanoporous structure largely increases the surface area of the membrane and thus the capacitance. The presence of the macroporous structure reduces the stiffness of the carbon membrane and improves its compliance with soft biological interfaces. Further microfabrication was performed on the porous carbon film to give the interdigital microsupercapacitor shown in Figure 4a ii. The bioelectronic stimulation of the device was evaluated on a rat heart (Figure 4a iv). Upon stimulation, the heart immediately contracted at double the stimulation rate, demonstrating the functionality of the device (Figure 4b).

Intrinsically stretchable organic electronics are an emerging candidate to replace traditional rigid electronics that are not compliant to soft biological tissues.^53^ Being flexible, stretchable and conductive, poly(3,4-ethylenedioxythiophene):polystyrenesulfonate (PEDOT:PSS) is widely used for bioelectronics devices.^54^ However, the immersion of PEDOT:PSS in an aqueous solution would wash non-cross-linked additives away and significantly drop the performance of the material. To enhance stretchability, stability, and conductivity, a cross-linkable supramolecular additive based on a polyrotaxane (PR) structure is used.^22^ The PR is composed of a poly(ethylene glycol) (PEG) backbone and sliding cyclodextrins (CDs) functionalized with PEG methacrylate (PEGMA) (Figure 4c). PEG induces the aggregation of PEDOT and replaces a portion of PSS (Figure 4d), while sliding CD units prevent the crystallization of PEG and improve the stretchability. The material is photopatternable, allowing for bioelectronics applications with high precision. The resulting material exhibits excellent conductivity even when stretched to 150% strain. A stretchable electrode array made from the modified PEDOT:PSS forms an intimate contact with the fourth ventricle of a rat (Figure 4e). A current pulse is delivered to individual electrodes to stimulate the tongue, whiskers, and neck separately, while EMG and motion signals at those locations are simultaneously recorded. Distinct muscle electrophysiological signals and movements are elicited by the stimulation of each electrode (Figure 4f). An activation map is further constructed by normalizing the EMG signal, showing the spatial distribution of different nuclei that downstream are connected with the hypoglossal nerve, facial nerve, and accessory nerve. The electrode array also shows less tissue damage or inflammatory responses compared with rigid plastic probes supported on polyimide substrates.

Macroscopic supramolecular hydrogels have attracted great interest in tissue scaffolding, diagnostics, and drug delivery due to their biocompatibility and stimuli-responsive properties.^55^ These hydrogels are produced when molecules are held together spontaneously by dynamic noncovalent interactions, such as hydrogen bonding, van der Waals forces, and π–π stacking.^56^ One of the key advantages of macroscopic supramolecular hydrogels is their ability to mimic the natural extracellular matrix (ECM) of living tissues. The ECM is a complex network of macromolecules that provides structural support and biochemical cues for cells to function properly. By designing hydrogels that mimic the ECM, it is possible to create scaffolds that can support cell growth, proliferation, and differentiation.^57^ In addition, macroscopic supramolecular hydrogels can be loaded with bioactive molecules, such as therapeutic proteins, that can promote specific cellular responses.^58^ This makes them promising candidates for use in tissue engineering, wound healing, and regenerative medicine.

Monolithic block copolymers feature other macroscopic self-assembled materials. Diblock copolymers can self-assemble into a variety of structures, including body-centered-cubic spheres, hexagonally packed cylinders, bicontinuous gyroids, and lamellae depending on the chemical nature of the polymer blocks.^59^ These ordered structures give rise to unique physical and chemical properties that make block copolymers useful in bioelectronics. To ensure an intimate skin interface and stable electrical communication between electronics and tissue through the soft hydrogel, the interface between tissue and hydrogel is expected to have tunable adhesion.^60^ Good adhesion is needed during therapy to ensure seamless contact while weak adhesion is desired when removing hydrogel from the skin to mitigate secondary damage to delicate tissue and prevent a commonly occurring skin condition known as medical adhesive-related skin injury.^60,61^ To achieve the goal, an interpenetrated double-network structure is synthesized through in situ radical polymerization of a thermally responsive covalent network of *N*-isopropylacrylamide (NIPAM) and acrylamide (AAm) in the presence of a physically cross-linked conducting polymer network of PEDOT:PSS.^60^ The resulting hydrogel shows a low contact impedance and high toughness. It also shows great adhesion with tissue below a lower critical solution temperature (LCST). While the temperature is above the LCST, the backbone aggregates, leaving less effective bonding sites with the external surface and making the hydrogel easy to detach from the tissue.

## Outlook

**Figure 5:**
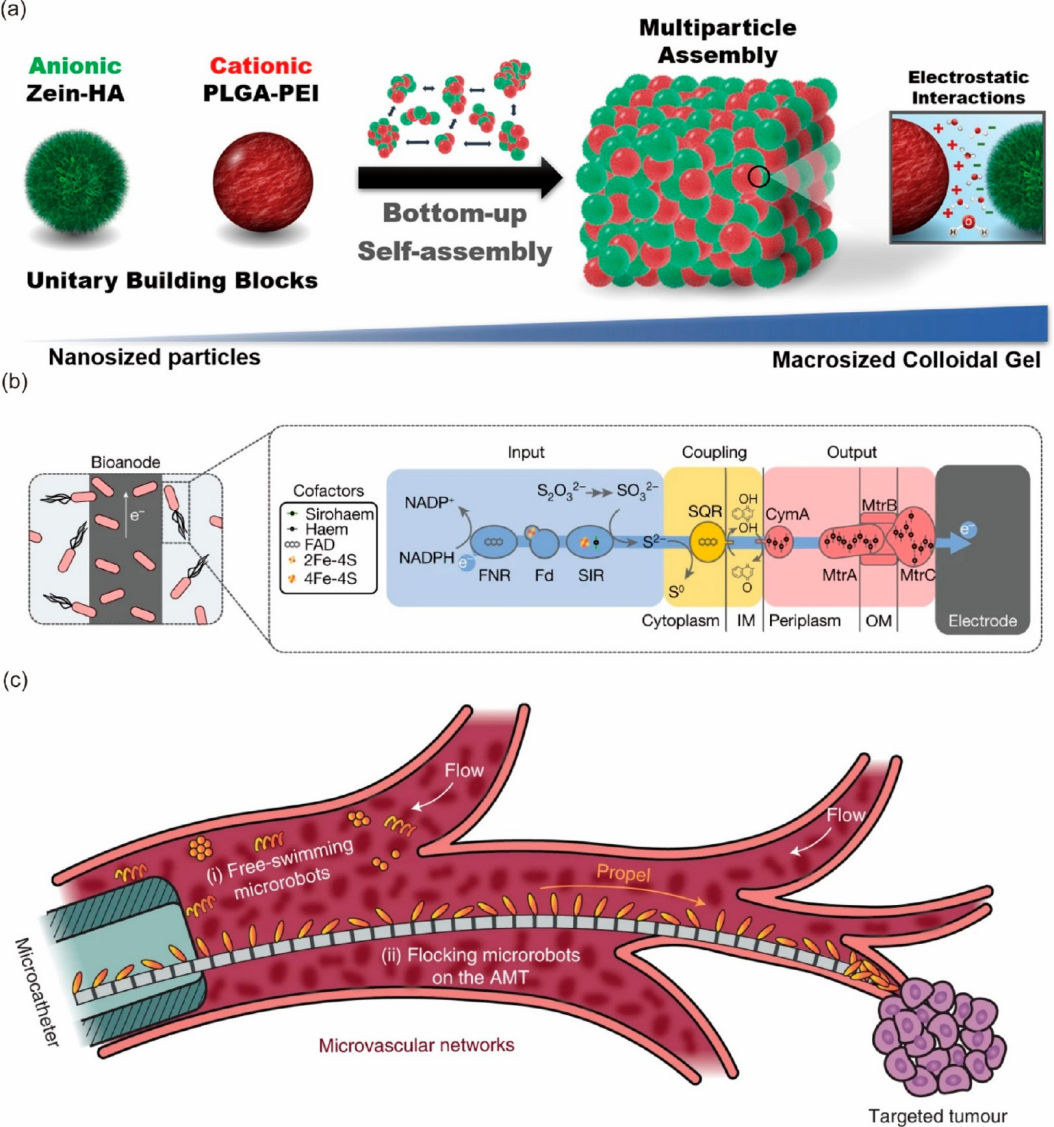
Outlook for spatial imaging and localized drug delivery, functionality, and colloidal manipulation. (a) Nanoscale building blocks are combined into higher-order multiparticle assemblies, ultimately producing macroscopic colloidal gel platforms. Imaging nanoparticles and therapy nanoparticles can be incorporated to achieve spatial imaging and localized drug delivery simultaneously. Panel a is adapted from ref (64). Copyright 2020 American Chemical Society. (b) An *E. coli* sensor with a synthetic electron transfer chain. Panel b is adapted with permission from ref (69). Published 2022 by Springer Nature. (c) A graphical illustration of artificial microtubule (AMT) deployment in microvascular networks. Panel c is adapted with permission from ref (72). Published 2022 by Springer Nature.

Despite advances in the field, there are still plenty of limitations when it comes to colloid-based materials including spatial imaging and localized drug delivery, functionality, and colloidal manipulation. New imaging techniques have been created that can potentially increase imaging qualities. For example, near-infrared luminescence has been shown to be a more accurate, cheaper, and safer alternative to other conventional imaging techniques, as near-infrared luminescence benefits from decreased photon scattering and less absorption within biological tissues, allowing for deeper optical penetration and higher image resolution.^62^ When injected into the body, fluorophore particles with different sizes (from a few nanometers to more than 10 μm) face different biological barriers and accumulate at different locations. To achieve luminescence imaging with high contrast, site-specific delivery of fluorescent probes is desired, together with a long circulation time, low nonspecific deposition, and high accumulation at target sites, requiring further fluorophore optimization.^62^ Also, improvements to magnetic resonance imaging (MRI) have been made via the self-assembly of MNPs under the assistance of polymer. The combination of the polymer and MNPs offers unique advances in medical diagnosis and treatment. Polymer-assisted MNP imaging has shown advantages in control over the assembly, disassembly, and stability of these MNPs in various biological environments. Polymer-assisted MNPs also allow for multimodal imaging, as multiple functional groups can be added onto the polymer network.^63^ Under the strides in imaging techniques, colloidal systems can be used to improve spatial controllability for drug release. For example, two complementary nanosized building blocks have been shown to produce biocompatible, injectable biomaterials (Figure 5a). These materials are highly stable and allow for localized multiparticle delivery, conferring a wide range of biomedical applications.^64^ Incorporating both imaging nanoparticles, such as MNPs, and therapy nanoparticles into colloidal gels through a bottom-up self-assembly method can decrease the invasiveness and negative biological response of the imaging nanoparticles and allow for simultaneous treatment. For example, if a clinician suspected a tumor in the brain, then a colloidal gel consisting of pH-sensitive MNPs as well as antioncological drugs could be used to identify where the tumor is present and release the therapeutic drugs through external stimulation at the specific location. The same combination could be used with colloidal gels and near-infrared fluorophores, unlocking more biomedical applications. Colloidal gels could also incorporate new drug delivery methods that capitalize on spatial control. For instance, the nanotopographical design of a biointerface has shown new functionality for drug delivery, including improved bioadhesion, targeted cellular uptake, and increased drug distribution.^65^ This drug delivery technique could be combined with colloidal gels, which could improve the spatial control of the nanotopographical drug release.

Along with spatial imaging and localized drug delivery, colloidal systems could gain new functionality by incorporating synthetic colloid-based materials into naturally occurring biomaterials. All of the biomaterials derived from living creatures, such as bacteria, plants, and animals, can be a good scaffold for harboring functional synthetic materials, on the one hand, to increase biocompatibility and bypass inflammation and, on the other hand, to introduce new functionality and enhance controllability. For example, bacterial cellulose-based composite scaffolds can embed various other materials, such as polymers and nanoparticles, and have shown many biomedical applications, including wound healing, bond tissue engineering, new cancer therapies, etc.^66^ Another strategy to introduce new functionality is to harbor living cells by scaffolds and make engineered living materials, which could be applied in various biomedical applications, including biosensing, stem-cell-based tissue engineering, and drug delivery.^67^ Inorganic particles or electronic devices can be integrated either as a signal generator to stimulate cells to release chemicals and kill cells to ensure biosafety or as a readout to sense and amplify the biological change. For those bioelectronic devices, tissue-like properties are desired to minimize the mechanical mismatch from real tissues so as to greatly maintain cell activities and ensure better communication.^68^ New functionality could be further raised by genetically engineering the living components and the corresponding nonliving environment. For example, a new method in bioelectronic sensing has been established through the programming of certain strains of *E. coli* and the manipulation of the electron transport chain (Figure 5b).^69^ The resulting system can be useful in environmental sensing and regulation and human health monitoring and regulation.

The development of advanced colloidal manipulation techniques holds great promise for improving biomedical applications, especially in the areas of minimally invasive surgery and precision drug delivery. Researchers are actively exploring new approaches to overcome the challenges of delivering colloidal materials into the human body with greater speed, precision, and control. One promising approach is the use of micro- and nanorobots, which can navigate through the body’s viscous fluids and precisely target specific sites.^70^ These microrobots can be propelled and controlled through a range of mechanisms, such as magnetic fields, acoustic waves, electrical fields, and light.^71^ However, significant challenges remain in developing microrobotics technology that can effectively navigate and disperse in the complex and dynamic flow environments of the body.^72^ Researchers are exploring a range of approaches, such as designing robots with more efficient propulsion mechanisms,^73^ improving their control systems via machine learning,^71^ and optimizing their surface chemistry^74^ to better interact with biological environments. One of the approaches is to use tubular medical catheters to direct the functional microrobot suspensions to the tip position.^75^ While this method shows promise, it is limited by the difficulty of miniaturizing catheters to the micrometer scale, as pumping pressure increases significantly with decreasing diameter. To overcome this limitation, researchers developed an artificial microtubule with embedded micromagnets that serve as stepping stones to guide particles rapidly through flow networks (Figure 5c).^72^ Such a new method requires further biomedical tests to demonstrate its functionality in biological systems. The field of colloidal manipulation is rapidly advancing, holding great promise for miniinvasive and precise biomedical applications, and is likely to have a significant impact on the field of medicine in the coming years.
